# Bai-He-Gu-Jin-Tang formula suppresses lung cancer via AKT/GSK3β/β-catenin and induces autophagy via the AMPK/mTORC1/ULK1 signaling pathway

**DOI:** 10.7150/jca.62779

**Published:** 2021-09-09

**Authors:** Quhui Wu, Da Li, Taoli Sun, Jian Liu, Huiping Ou, Lei Zheng, Xuyang Hou, Wenqun Li, Fuyuan Fan

**Affiliations:** 1 Department of Respiratory Medicine, The First Affiliated Hospital of Hunan University of Chinese Medicine, Changsha, China.; 2 Department of Pharmacy, The Second Xiangya Hospital, Central South University, Changsha, 410011, China.; 3 Medical School, Hunan University of Chinese Medicine, Changsha, 410208, P. R. China.

**Keywords:** Bai-He-Gu-Jin-Tang, Complementary and alternative treatment, Lung cancer, autophagy, apoptosis

## Abstract

**Aims:** Bai-He-Gu-Jin-Tang (BHGJT) is a classic Chinese formula used to treat lung cancer, while the underlying molecular mechanism remains obscure. The aim of the study was to investigate the molecular mechanism of BHGJT on lung cancer and demonstrate the potential for synergistic treatment combining BHGJT with conventional therapy.

**Methods:** Cell viability assay, colony formation assay and EdU assay were used to determine the *in vitro* effects of BHGJT, and a subcutaneous xenograft model was used to evaluate the *in vivo* effect. Cell cycle analysis, apoptosis rate analysis, immunohistochemical and immunofluorescent staining, Western blot assays and network pharmacology-based analysis were used to explore the underlying mechanisms.

**Results:** We found that BHGJT inhibited cell proliferation via a dose-dependent pathway and obviously hindered tumor growth *in vivo* in lung cancer. Cell cycle arrest and apoptosis were pronouncedly induced by BHGJT via dysregulation of the cell cycle regulators CDK4 and Cyclin D1 and dysregulation of apoptosis-associated proteins, such as cleaved caspase 3/9 and the BCL-2 family. Based on a network pharmacology-based analysis and experimental evidence, we demonstrated that the AKT/GSK3β/β-catenin signaling pathways were responsible for BHGJT-induced apoptosis in lung cancer cells. Additionally, autophagy was induced by BHGJT via the AMPK/mTORC1/ULK1 signaling pathway, and blocking autophagy with either chloroquine or a ULK1 inhibitor increased the killing efficiency of BHGJT in lung cancer cells.

**Conclusion:** Our findings indicate that the BHGJT formula efficiently inhibits lung cancer growth and represents a potential complementary and alternative treatment for lung cancer.

## Introduction

Lung cancer is a common malignant disease with the highest incidence and mortality rate worldwide 1, and it is roughly divided into two categories: non-small cell lung cancer (NSCLC), which accounts for approximately 80% of cases, and small cell lung cancer. In recent decades, the treatments of lung cancer have advanced, and various cutting-edge therapies, such as neoadjuvant chemotherapy, targeted therapy and immunotherapy, may increase the survival of patients with lung cancer 2-4. However, the overall efficiency remains unsatisfactory. Traditional Chinese medicines (TCMs) exhibit robust anticancer effects and present advantages that include improving the quality of life, reducing adverse effects caused by conventional therapies, and augmenting synergistic efficiency in the treatment of cancer 5. Evaluating the anticancer effects of TCM and revealing the underlying molecular mechanisms using modern techniques have been increasingly recognized as important research topics.

Bai-He-Gu-Jin-Tang (BHGJT) is a classic Chinese formula for protecting lung function and treating lung discomfort and one of the most commonly used formulas in China for patients with lung cancer 6, 7. Herbs prescribed in TCM represent four categories for the treatment of one disease: “Jun” means the major herbs and includes Bulbus Lilii, Radix Rehmanniae, and Radix Rehmanniae Preparata; “Chen” plays a minor role and includes Radix Ophiopogonis and Radix Scrophulariae; “Zuo” plays a helper role and includes Bulbus Fritillariae Cirrhosae, Radix Platycodi, Radix Angelicae Sinensi and Radix Paeoniae Alba; and “Shi” represents additive functions and includes Radix Glycyrrhizae Preparata. Clinical studies have demonstrated that the BHGJT formula contributes to decreasing the mortality of lung cancer 6. The combination of BHGJT with chemotherapy significantly improved the efficiency and decreased the side effects in patients with lung cancer compared to the chemotherapy group 8. Modern pharmacological studies revealed that polysaccharides derived from Bulbus Lilii pronouncedly inhibited the growth of melanoma and Lewis lung carcinoma in mice 9. Catalpol extracted from Rehmanniae suppressed tumor growth and invasion by inhibiting inflammation and tumor angiogenesis in colon cancer 10. In addition, 2,5-dihydroxyacetophenone (DHAP), an active compound from Radix rehmanniae preparata, significantly inhibited cell proliferation and induced apoptosis by activating MAPK signaling in cancer cells 11. Therefore, multiple lines of clinical and experimental evidence suggest that BHGJT is efficient in killing lung cancer or beneficial for lung cancer patients; however, the molecular evidence has not yet been obtained.

Induction of cell cycle arrest and programmed cell death is the principal therapeutic target for cancer 12. Unscheduled cell proliferation is one of the hallmarks of cancer 13. The therapeutic potential of targeting cyclin-dependent kinases, such as CDK4/6 and Cyclin D, has been recognized, and several inhibitors, such as palbociclib and ribociclib, have been approved for the treatment of lung cancer 14. Regarding apoptosis, various drugs designed to target intrinsic antiapoptotic genes, such as BCL-2, MCL1, or IAP, have been approved for clinical application 15. Additionally, autophagy, another form of programmed cell death, shows opposing and context-dependent roles in the regulation of cell fate; thus, deliberate manipulation of autophagy is required in clinical interventions 16. Therefore, it is reasonable and essential to reveal whether BHGJT inhibits lung cancer by regulating these bioprocesses and the underlying mechanisms.

In the current work, we demonstrated that BHGJT inhibited lung cancer growth by inducing cell cycle arrest via the downregulation of CDK4 and Cyclin D1 and apoptosis via the AKT/GSK3β/β-catenin signaling pathways. We also found that BHGJT induced marked autophagy through the AMPK/mTORC1/ULK1 signaling pathway and that blockade of autophagy further improved the efficiency of BHGJT in lung cancer cells. Our report provides evidence to support the molecular mechanisms underlying the effects of the BHGJT formula as an anti-lung cancer therapy and suggests that the combination of autophagy inhibitors with BHGJT might be a more robust remedy.

## Materials and methods

### BHGJT formula preparation

The BHGJT formula drug preparation consists of 10 commonly used Chinese herbs, including Bulbus Lilii, Radix Rehmanniae, Radix Rehmanniae Preparata, Radix Ophiopogonis, Radix Scrophulariae, Bulbus Fritillariae Cirrhosae, Radix Platycodi, Radix Angelicae Sinensi, Radix Paeoniae Alba and Radix Glycyrrhizae Preparata. The raw herbs for the preparation of BHGJT were purchased from The First Affiliated Hospital of Hunan University of Chinese Medicine and identified by two pharmacists. The ratio of these herbs (in grams) was as follows: 5:6:9:5:5:5:5:5:5:5. The pharmaceutical raw materials of BHGJT were smashed and soaked in 70% ethanol overnight and then centrifuged, and the residues were discarded. The supernatant was evaporated by low-temperature evaporation and desorption drying until a semisolid state of BHGJT (extractum) is formed. The drug was stored at -20 °C until further use.

### Cell lines and culture

The lung cancer cell lines A549 and H1299 were purchased from ZSBIO, China, and genotyped via STR by the same company. A549 cells were cultured in DMEM/F12-Dulbecco's modified Eagle's medium supplemented with 10% fetal bovine serum and 1% penicillin-streptomycin. H1299 cells were cultured in RPMI-1640 medium supplemented with 10% fetal bovine serum and 1% penicillin-streptomycin. All cells were incubated in a humidified incubator at 37°C and 5% CO2.

### Cell viability detection

Cells in logarithmic growth phase were trypsinized and seeded into 96-well plates. After incubation overnight, various concentrations of BHGJT (0, 0.5, 1, 2, 4, and 8 mg/ml) were added to 96-well plates and cultured for 48 h. A CCK-8 kit (GenView, China) was used to determine cell viability. The OD value was measured at a wavelength of 450 nm by a microplate spectrophotometer (Thermo Fisher, America). The experiment was repeated three times. All values were normalized to the control group (without adding cells), and the results are presented as the mean ± SD.

### Colony formation assay

Cells in logarithmic growth phase were trypsinized, seeded into 6-well plates at 5000 cells per well, and cultured for 3 days. Then, BHGJT was added as indicated, followed by another culture period of 4 days. Subsequently, the supernatant was discarded, the cells were washed two times with PBS, and the colony was then fixed with 4% paraformaldehyde. Then, the cells were stained with 1% crystal violet (Beyotime, China), and colony images were captured with a digital camera.

### EdU assay

H1299 and A549 cells were seeded into 24-well plates and cultured overnight. BHGJT was added as indicated and cultured for 48 h. EdU working solution was prepared and added to the plates for culture for 3 hours. Then, the supernatant was discarded, the cells were washed two times with PBS, the cells were fixed with 4% paraformaldehyde, and the cell membranes were lysed with 0.25% Triton X-100. Then, the Click Addictive Solution was prepared and added to the cells, which were cultured for 30 min without light. The cell nucleus was stained with DAPI, and the EdU signal was determined by a fluorescence microscope (Olympus Inc., America).

### Cell cycle analysis

The cell cycle distribution was analyzed by a Cell Cycle Analysis Kit purchased from Genview, China, and used according to the manufacturer's guidelines. Briefly, cells were harvested, washed three times with PBS, resuspended in 1× binding buffer and stained with PI solution after fixation with 70% alcohol for 2 hours at 37 °C without light. Next, the cells were sent for testing via a BD FACSARIA II flow cytometry (Becton Dickinson, USA).

### Subcutaneous xenograft model

BALB/c nude mice received human care in compliance with the guidelines implemented at Second Xiangya Hospital, Central South University. The study was performed according to international, national and institutional rules for animal experiments and biodiversity rights. Briefly, 2 × 10^6^ A549 cells were subcutaneously injected into the right dorsal region of 6-week-old male nude mice (n=5-6). BHGJT was intragastrically administered to mice from the first day at a dose of 400 or 800 mg/kg body weight per day, and saline served as a control. The tumor sizes were measured every 5 days. After 4 weeks, the mice were sacrificed and the tumors were collected and fixed with 4% paraformaldehyde.

### Immunohistochemical staining (IHC)

Antibodies against Ki67 and cleaved caspase 3 were purchased from Abclonal, China and CST, America, respectively. The IHC kit was provided by ZSGB-BIO, China, and used according to the manufacturer's guidelines. Briefly, the sections were heated to deparaffinization and incubated with 3% H_2_O_2_ for the proper time. Epitope retrieval was performed by heating in sodium citrate buffer at 96 °C for 30 min. For antigen-antibody reaction, samples were incubated with rabbit anti-human Ki67 (1:100 dilution) and cleaved caspase 3 (1:1000 dilution) primary antibodies for 2 hours. The cells were washed with PBS and incubated with secondary antibody followed by DAB staining and hematoxylin counterstaining. The sections were dehydrated, soaked in xylene, mounted with neutral balsam and further fastened by enamel.

### Western blot analysis

Lung cancer cells were lysed in RIPA buffer supplemented with protease and phosphatase inhibitors (TargetMol, USA) and incubated on ice for 30 min. The supernatant was collected after discarding the sedimentation. The denatured proteins were added to the chamber for SDS-PAGE, followed by electrotransfer onto PVDF. After blocking with 3% BSA for 1 h, the membranes were incubated with diluted primary antibody overnight at 4 °C. On the following day, the primary antibody was discarded and the membranes were washed three times with TBST, followed by incubation with diluted secondary antibody for 1 h at room temperature. The immune complexes were detected via an enhanced chemiluminescence system (Life Tec, USA). Analysis and quantification of the bands were performed using ImageJ software (Version 11). The primary antibodies involved in this manuscript included the following: CDK4 (1:1000; Abclonal, China), Cyclin D1 (1:1000; Abclonal, China), cleaved caspase 3 (1:1000; CST, America), caspase 3 (1:1000; Abclonal, China), caspase 9 (1:1000; Abclonal, China), cleaved caspase 9 (1:1000; Abclonal, China), Bad (1:1000; Abclonal, China), Bax (1:1000; Abclonal, China), LC3 (1:1000; CST, America), GSK3β (1:1000; Abclonal, China), p-GSK3β (1:1000; Abclonal, China), AKT (1:1000; CST, America), p-AKT (1:1000; CST, America), BCL-2 (1:1000; Abclonal, China), β-catenin (1:1000; Abclonal, China), Tubulin (1:1000; Abclonal, China), Lamin A/C (1:1000; Abclonal, China), AMPK (1:1000; CST, America), p-AMPK (1:1000; CST, America), p-p70 S6 (1:1000; Abclonal, China), p-ULK1 S555 (1:1000; CST, America) and ULK (1:1000; CST, America). Secondary antibodies were also purchased from Abclonal, China.

### Flow cytometry assay for apoptosis

To quantitively detect the apoptosis rate of lung cancer cells upon treatment with BHGJT, a flow cytometry assay was performed using an Annexin V-FITC/PI apoptosis detection kit (Genview, China). Cells in each group were treated as indicated. Then, the cells were collected, washed with PBS, resuspended in 100 μL binding buffer containing 5 μL Annexin V-FITC and 10 μL PI staining solution, and incubated in the dark at room temperature for 10 minutes. Then, 400 μL binding buffer was added to each sample and fully mixed. The apoptosis rate was analyzed by flow cytometry (Becton Dickinson, America).

### Immunofluorescent staining of LC3

Immunofluorescent staining was performed on 6 μm paraffin sections. The sections were deparaffinized and incubated with 3% H_2_O_2_ in the dark for 15 min, followed by epitope retrieval with sodium citrate buffer (10 mM sodium citrate and 0.05% Tween 20 at pH 6.0) at 96°C for 30 min. Then, the sections were blocked with 5% goat serum (16210064, Fisher Scientific, America). Anti-LC3 (CST, America) was added to the sections and incubated overnight. The next day, the cells were washed three times with PBST, and the anti-rabbit secondary antibody was added and incubated for 1 h at room temperature. Nuclei were stained with DAPI containing antifade reagent (P36935, Invitrogen, America). Images were acquired using an Olympus fluorescence microscope.

### High-performance liquid chromatography (HPLC) analysis of BHGJT

HPLC analysis was performed according to a previous report 17, using a Shimadzu LC-20AT HPLC (Shimadzu Corporation) equipped with a degasser, binary pump, thermostatted column oven, autosampler and photodiode array detector. The BHGJT sample was separated on a Kromasile-C18 column (4.6 × 250 mm, 5 µm) at 30 °C. The mobile phase system was composed of (A) water and (B) methanol. The HPLC gradient elution profile was as follows: 0~60 min, 5%~95% B. The mobile phase flow rate was 1.0 ml/min, and the UV spectrum was 260 nm. The injection volume was 20 μL. Data acquisition was performed by Shimadzu LC solution software (Shimadzu Corporation).

### Nuclear and cytoplasmic extraction

An extraction kit was purchased from Fisher Scientific, America, and manipulated according to the instructions. Briefly, cells were collected by centrifugation at 500 × g for 5 min, the supernatant was removed, ice-cold CER I was added to the cell pellet, and the cells were vortexed vigorously. After incubation for 10 min, ice-cold CER II was added to the tube, which was centrifuged at 16000 × g for 5 min to obtain the cytoplasmic extract. The pellet was resuspended in ice-cold NER, incubated for 40 min, and then centrifuged at maximum speed for 10 min to obtain the nuclear extract.

### Network pharmacology-based analysis

The target genes of lung cancer were retrieved from the TTD (http://db.idrblab.net/ttd), DrugBank (https://www.drugbank.ca), Disgenet (http://www.disgenet.org) and pharmGKB (https://www.pharmgkb.org) databases. The top 400 genes in the Disgenet database according to DSI and score were selected. A total of 514 target genes of lung cancer were obtained. The active components in BHGJT were retrieved from the TCMSP (http://lsp.nwu.edu.cn/tcmsp.php) and TCMID databases (https://bigd.big.ac.cn/databasecommons). After screening by cutoff values of OB (bioavailability) ≥ 30% and DL (drug likeness) ≥ 0.18 and removing the overlapping components, a total of 135 components in BHGJT were obtained. The target proteins of the bioactive components of BHGJT were retrieved from the TCMSP database and TCMID based on the SwissTargetPrediction platform. Then, we constructed the compound-target network of BHGJT in lung cancer. GO and pathway enrichment analyses of the involved targets were conducted via R version 4.0.4.

### Statistical analysis

All data are expressed as the means ± standard error using GraphPad Prism 8. Animal experiments were performed at least 5 times, and cell experiments were performed at least 3 times. Unpaired Student's t tests were used to evaluate the significance between 2 groups, and one-way analysis of variance was used to determine the differences among multiple groups. A value of p<0.05 was regarded as statistically significant.

## Results

### BHGJT inhibited tumor growth *in vitro* and *in vivo*

**Figure 1:**
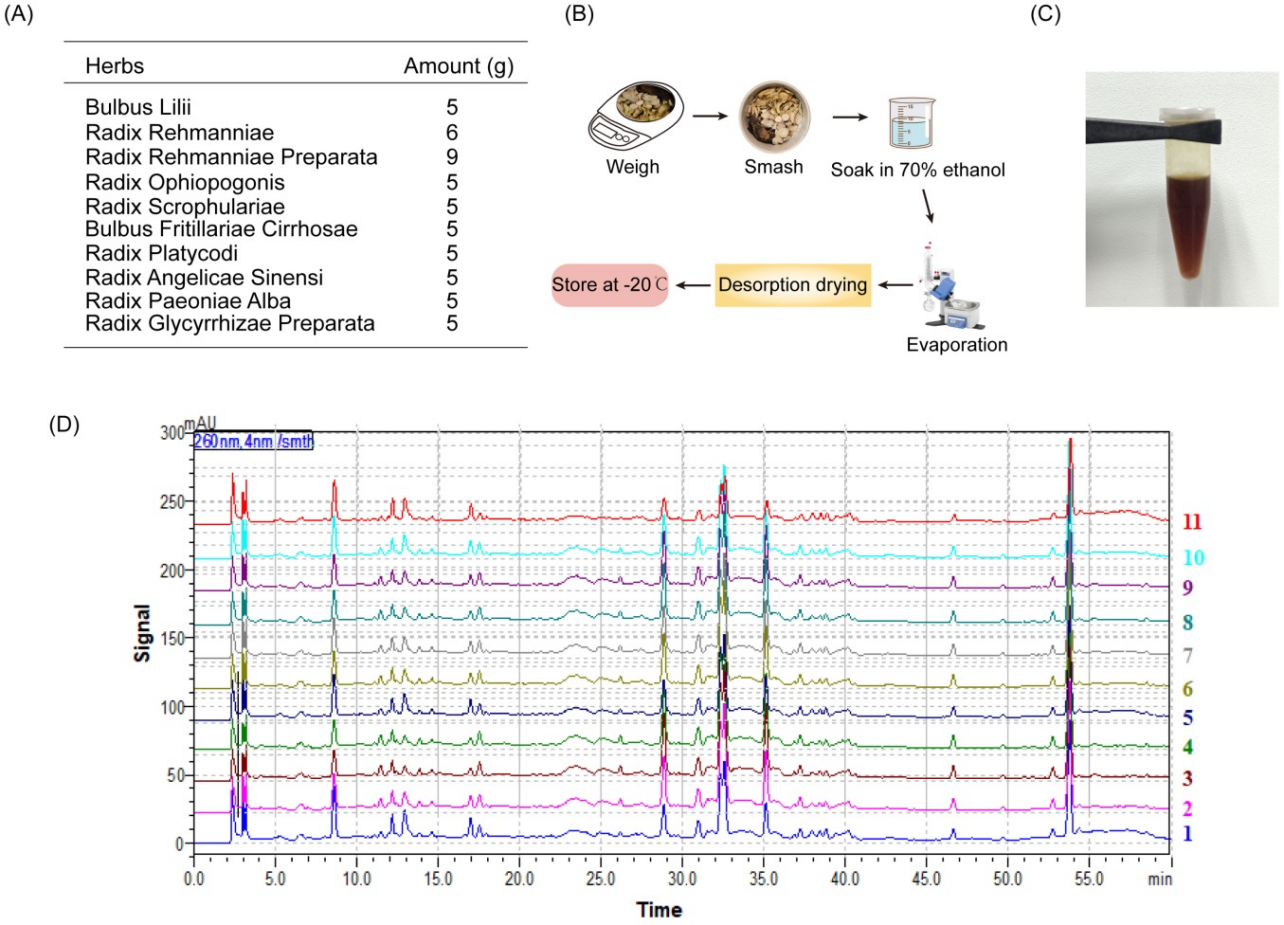
** BHGJT preparation. (A)** The composition of BHGJT. **(B)** A schematic of the method of preparation. **(C)** BHGJT solved in PBS. **(D)** HPLC analysis of BHGJT, the comparison of 11 different times BHGJT (No.:1-11).

**Figure 2:**
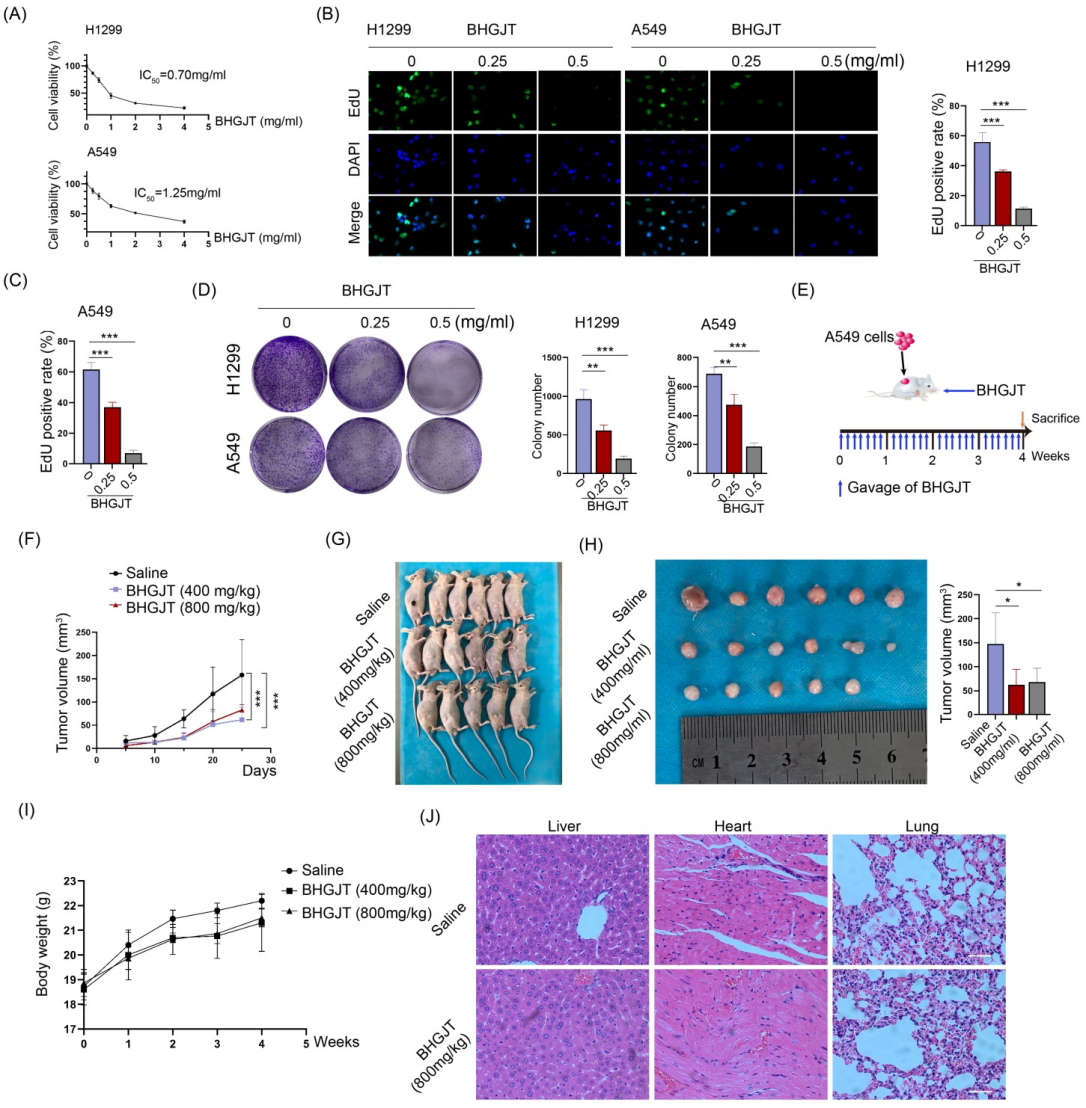
** BHGJT inhibited tumor growth *in vitro* and *in vivo*. (A)** H1299 and A549 cells were seeded into 96-well plates overnight. BHGJT was added and cultured for 48 h. Cell viability was detected by a CCK-8 kit, and the IC_50_ values were 0.70 and 1.25 mg/ml for H1299 and A549 cells, respectively. **(B)** EdU assay was used to detect the proliferation variation induced by BHGJT at doses of 0.25 and 0.5 mg/ml (left). In 1299 cells, BHGJT inhibited the proliferation rate by a dose-dependent pathway (right). **(C)** In A549 cells, BHGJT inhibited the proliferation rate by a dose-dependent pathway. **(D)** Cells were seeded in 6-well plates at 5000/well and cultured for 3 days, and then BHGJT (0.25, 0.5 mg/ml) was added for another 4 days (left). BHGJT inhibited colony formation by a dose-dependent pathway (right). **(E)** Schematic of BHGJT administration in mice; BHGJT was administered at 400 and 800 mg/kg body weight, and saline served as a control. **(F)** From day 5 after injection of cancer cells, the tumor volume was detected every 5 days. **(G)** Mice were sacrificed 28 days after injection of cancer cells. **(H)** Tumors were collected, and tumor volume was detected. BHGJT significantly inhibited tumor volume compared to the control. **(I)** The difference of body weight between BHGJT and saline group was not significant. **(J)** H&E staining of liver, heart and lung tissues showed no obvious injury was induced by BHGJT in mice. The scale indicates 100 µm; *p<0.05; ***p<0.001.

The drug preparation of BHGJT was based on Sheng-Zhai-Yi-Shu, a classic Traditional Chinese Medicine book. Drug was prepared as indicated (Figure 1A-C). Quality control was performed by HPLC analysis, and it showed stable and good repeatability of the drugs in 11 different batches (Figure 1D). Cell viability was analyzed by CCK-8 assay. The IC_50_ of BHGJT was 0.70 mg/ml and 1.25 mg/ml for H1299 and A549 cells after incubation for 48 h, respectively (Figure 2A). EdU assays showed that BHGJT significantly inhibited the proliferation of H1299 and A549 cells in a concentration-dependent manner (Figure 2B,C). For the colony formation assay, BHGJT significantly decreased the number of colonies after incubation for 7 days in H1299 and A549 cells at both tested concentrations (Figure 2D). To examine whether BHGJT could attenuate tumor growth *in vivo*, we administered BHGJT by gavage at 400 mg/kg and 800 mg/kg in a subcutaneous xenograft model. The therapy period of BHGJT was 4 weeks with consecutive gavage every day and is illustrated in Figure 2E. The tumor volume was measured every 5 days. Compared to the saline group, BHGJT significantly decreased tumor volume after therapy for 10 days and the difference of efficacy between the treatment groups was very slight, suggesting that gavage with 400 mg/kg BHGJT could induce a pronounced effect on lung cancer in an *in vivo* model (Figure 2F-H). Furthermore, the safety evaluation of BHGJT showed that it would not significantly injury the major organs in mice (Figure 2I,J). Thus, these data showed that BHGJT inhibited the proliferation of lung cancer cells and tumor growth *in vivo*.

### BHGJT induces G0/G1 cell cycle arrest and apoptosis in lung cancer *in vitro* and *in vivo*

**Figure 3:**
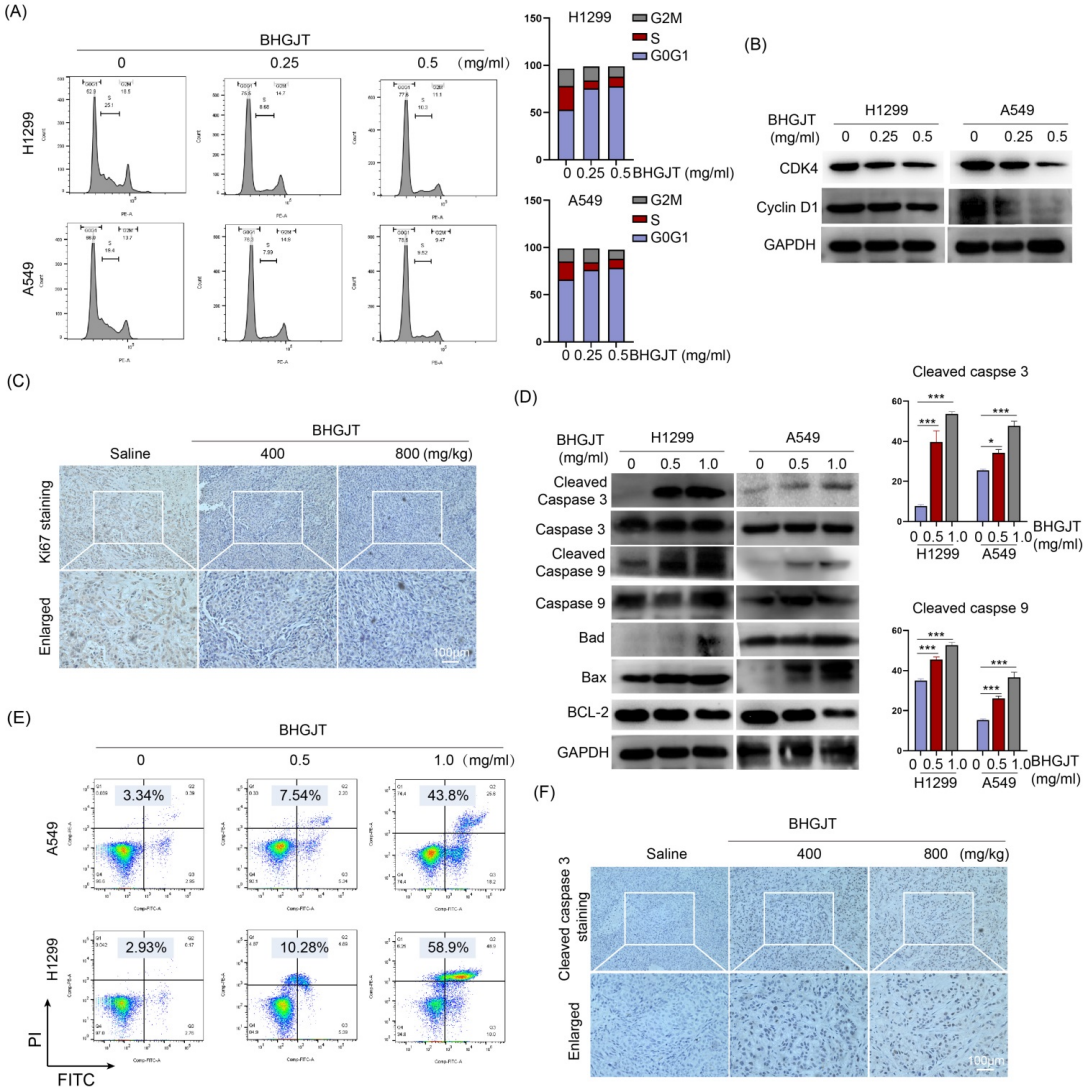
** BHGJT induced cell cycle arrest and apoptosis *in vitro* and *in vivo*. (A)** Cells were seeded in 6-cm dishes and cultured overnight. The next day, BHGJT was added and incubated for 48 h, and the cell cycle distribution was detected by flow cytometry. **(B)** BHGJT obviously decreased the expression of CDK4 and Cyclin D1 in H1299 and A549 cells upon BHGJT in a dose-dependent manner. **(C)** Immunohistochemical staining showed that BHGJT markedly decreased the expression of Ki67 in tumors from subcutaneous models. **(D)** Expression of apoptosis-associated proteins was detected, and BHGJT significantly increased the expression of cleaved caspase 3/9, Bad and Bax while decreasing BCL-2 expression in H1299 and A549 cells. **(E)** Apoptosis rate was quantitatively detected by flow cytometry. BHGJT (0.5 and 1.0 mg/ml) led to 7.54% and 43.8% apoptosis in A549 cells and 10.28% and 58.9% apoptosis in H1299 cells, respectively.** (F)** In samples from the subcutaneous model, cleaved caspase 3 was significantly elevated by BHGJT compared to the saline group. *p<0.05; ***p<0.001.

To further understand the mechanism of BHGJT-mediated effects on lung cancer, we examined whether it could influence the cell cycle distribution of lung cancer cells. As shown in Figure 3A, compared to the control group, BHGJT led to marked cell cycle arrest at G0/G1 in H1299 and A549 cells by a concentration-dependent pathway. Cyclin D1 accumulates and associates with CDK4 to phosphorylate the retinoblastoma protein, which generates the key regulator of G1 to S progression 18. Thus, we determined the expression of these cyclins. As expected, BHGJT markedly decreased the protein expression of CDK4 and Cyclin D1 in H1299 and A549 cells (Figure 3B). As the concentration increased, these cyclins were further inhibited by BHGJT in both cell lines. In line with these results, IHC staining showed that gavage with BHGJT (400 mg/kg, 800 mg/kg) pronouncedly inhibited Ki67 expression in tumor tissues compared to that of the control group (Figure 3C). Apoptosis is one of the main bioprocesses induced by toxic drugs in cancers. Cleaved caspase 3 and cleaved caspase 9 were elevated considerably in BHGJT-treated cells compared to the control cells in a concentration-dependent manner (Figure 3D). As the BCL-2 family contains protein factors that control cell survival and mitochondrial apoptosis, we detected the expression of Bax, BCL-2 and Bad in H1299 and A549 cells upon BHGJT treatment. Obvious upregulation of Bax and Bad and downregulation of BCL-2 were observed in the BHGJT-treated groups, suggesting that BHGJT induced apoptosis prominently through the mitochondrial pathway. Quantitatively, BHGJT (1.0 mg/ml) induced 43.8% apoptosis in A549 cells and 58.9% apoptosis in H1299 cells (Figure 3E). Furthermore, BHGJT led to marked expression of cleaved caspase 3 in tumor tissues based on IHC staining (Figure 3F). Taken together, these results indicated that G0/G1 cell cycle arrest and apoptosis were responsible for BHGJT-mediated tumor inhibition in lung cancer.

### Inhibition of autophagy facilitated the antitumor effect induced by BHGJT in lung cancer cells

**Figure 4:**
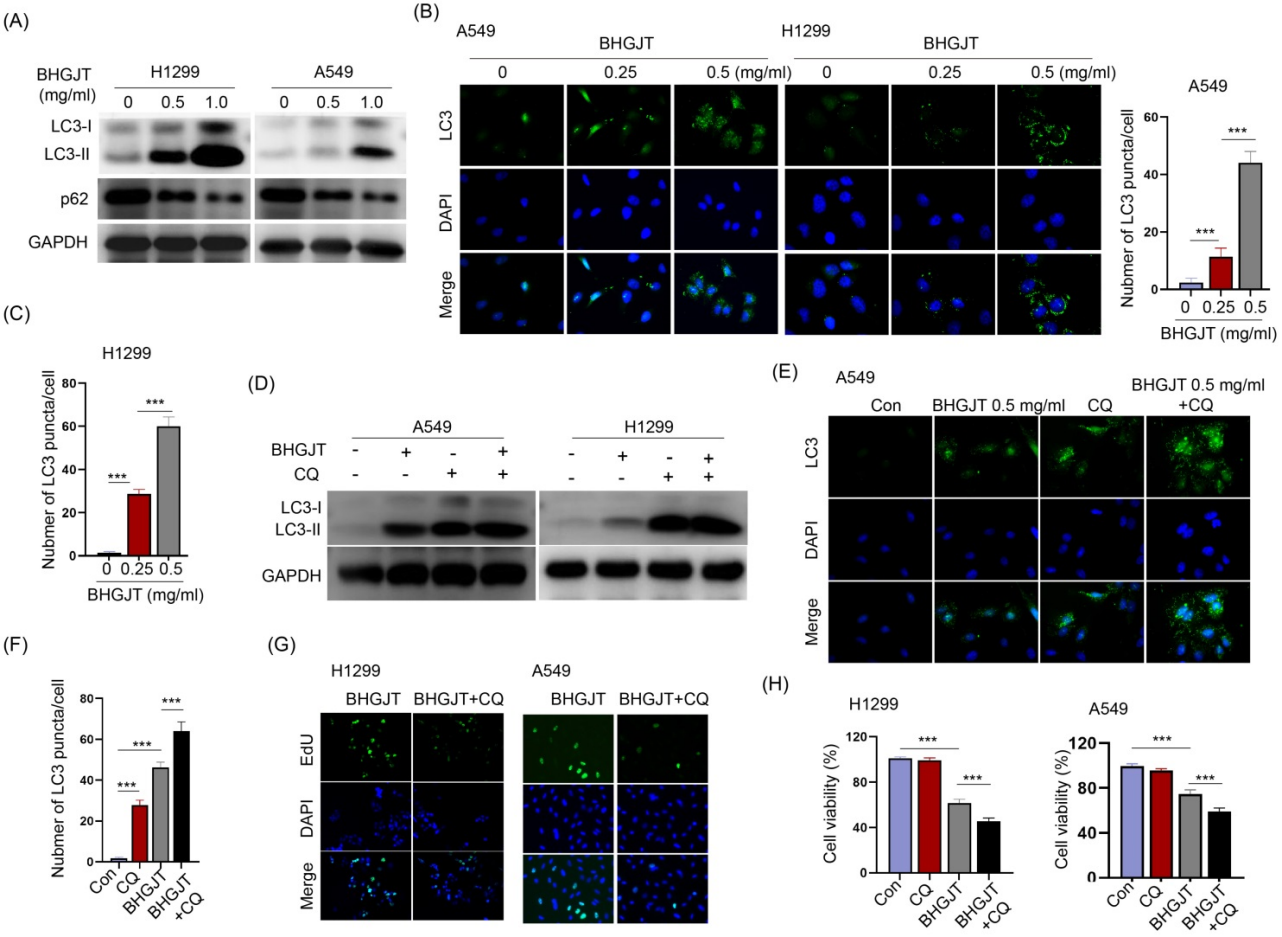
** BHGJT induced autophagy *in vitro*, and inhibiting autophagy augmented the efficiency. (A)** LC3-II is a marker of autophagy, and BHGJT markedly increased LC3-II expression but decreased p62 expression in H1299 and A549 cells. **(B,C)** Immunofluorescent staining of LC3 showed that BHGJT significantly increased the number of LC3 puncta in both cell lines. **(D)** CQ is an inhibitor of autophagy by neutralizing lysosomal pH. Cotreatment with CQ (10 µM) and BHGJT (0.5 mg/ml) increased LC3-II expression compared to that of the BHGJT group in both cell lines. **(E,F)** CQ inhibited autophagy by accumulating LC3 puncta in A549 cells. **(G)** In the presence of CQ, cell proliferation was significantly decreased upon stimulation with BHGJT, as analyzed by EdU assay in H1299 and A549 cells. (H) Cell viability was significantly decreased by CQ upon stimulation with BHGJT in H1299 and A549 cells. Con, control; **p<0.01; ***p<0.001.

Autophagy is a dual-edged sword in controlling cell survival and death. BHGJT markedly increased LC3-II expression upon BHGJT treatment in a concentration-dependent manner in H1299 and A549 cells (Figure 4A). LC3 puncta were significantly induced and accumulated in the cytoplasm by BHGJT. As the concentration of BHGJT increased, the number of LC3 puncta was further increased in both cell lines (Figure 4B,C). Chloroquine (CQ) is an inhibitor of autophagy that neutralizes lysosomal pH. As shown in Figure 4D, the expression of LC3-II was significantly increased after CQ treatment, and further increased by BHGJT in H1299 and A549 cells, suggesting that autophagy was obviously blocked by CQ upon stimulation with BHGJT. Immunofluorescence staining also showed the same trend as CQ in A549 cells (Figure 4E,F). We wanted to examine whether blocking autophagy influences the killing efficacy of BHGJT in lung cancer cells. The EdU assay showed that CQ significantly inhibited the proliferation of H1299 and A549 cells when coadministered with BHGJT (Figure 4G). Furthermore, CQ significantly reduced cell viability in the presence of BHGJT in H1299 and A549 cells (Figure 4H). Therefore, blocking autophagy could promote the inhibitory effects of BHGJT on lung cancer cells.

### BHGJT induces apoptosis by the AKT/GSK3β/β-catenin signaling pathway in lung cancer cells

**Figure 5:**
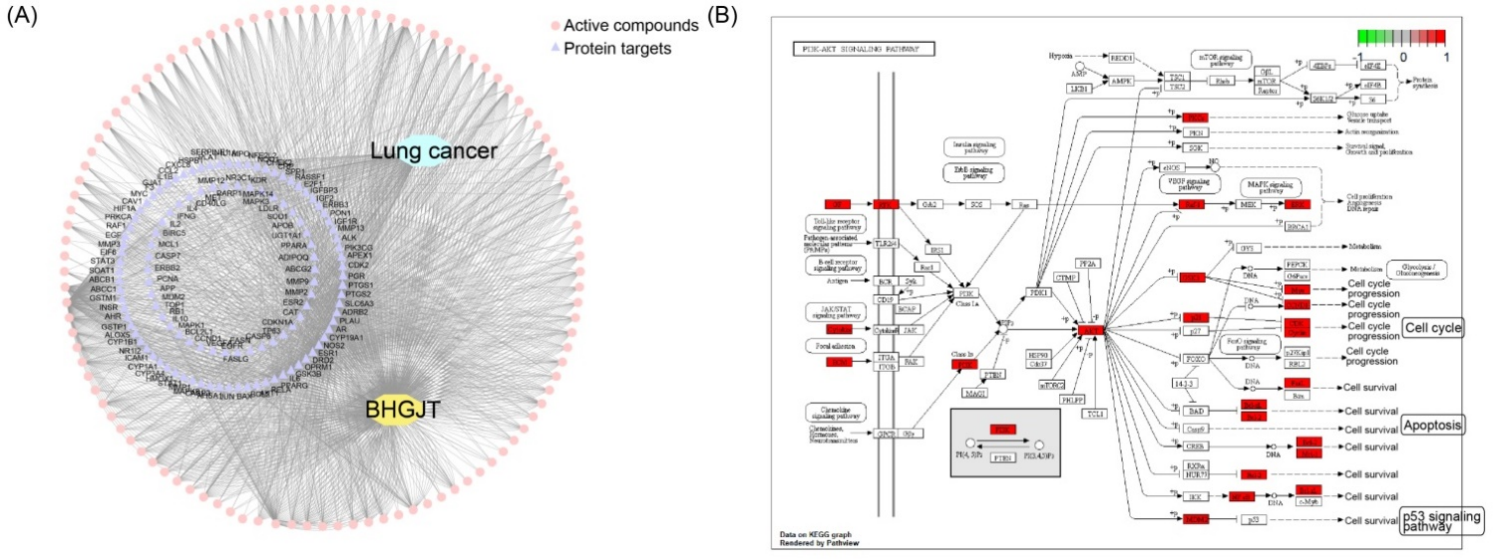
**Analysis based on the pharmacological network of BHGJT in lung cancer. (A)** Compound-target network for BHGJT in lung cancer. The pink nodes represent candidate active compounds, and the blue nodes represent potential protein targets. The edges represent the interactions between them, and lines are proportional to their degree. **(B)** KEGG analysis of the PI3K-AKT signaling pathway associated with BHGJT.

To reveal the molecular mechanism underlying the effect of BHGJT on lung cancer, we used computational tools and resources to investigate the pharmacological network of BHGJT in lung cancer. All components of the herbs involved in BHGJT were retrieved from TCMSP and TCMID. Lung cancer-related human genes were screened from the TTD, DrugBank, Disgenet, and pharmGKB databases. A total of 135 candidate bioactive components were obtained. Then, the targeted proteins of BHGJT were mapped with lung cancer-associated genes to construct the compound-target network of BHGJT in lung cancer (Figure 5A). The overlapping targets were used for pathway enrichment analysis. Among them, the PI3K-AKT signaling pathway was significantly enriched with the most targeted genes (Figure 5B), which was further examined by experiments.

**Figure 6:**
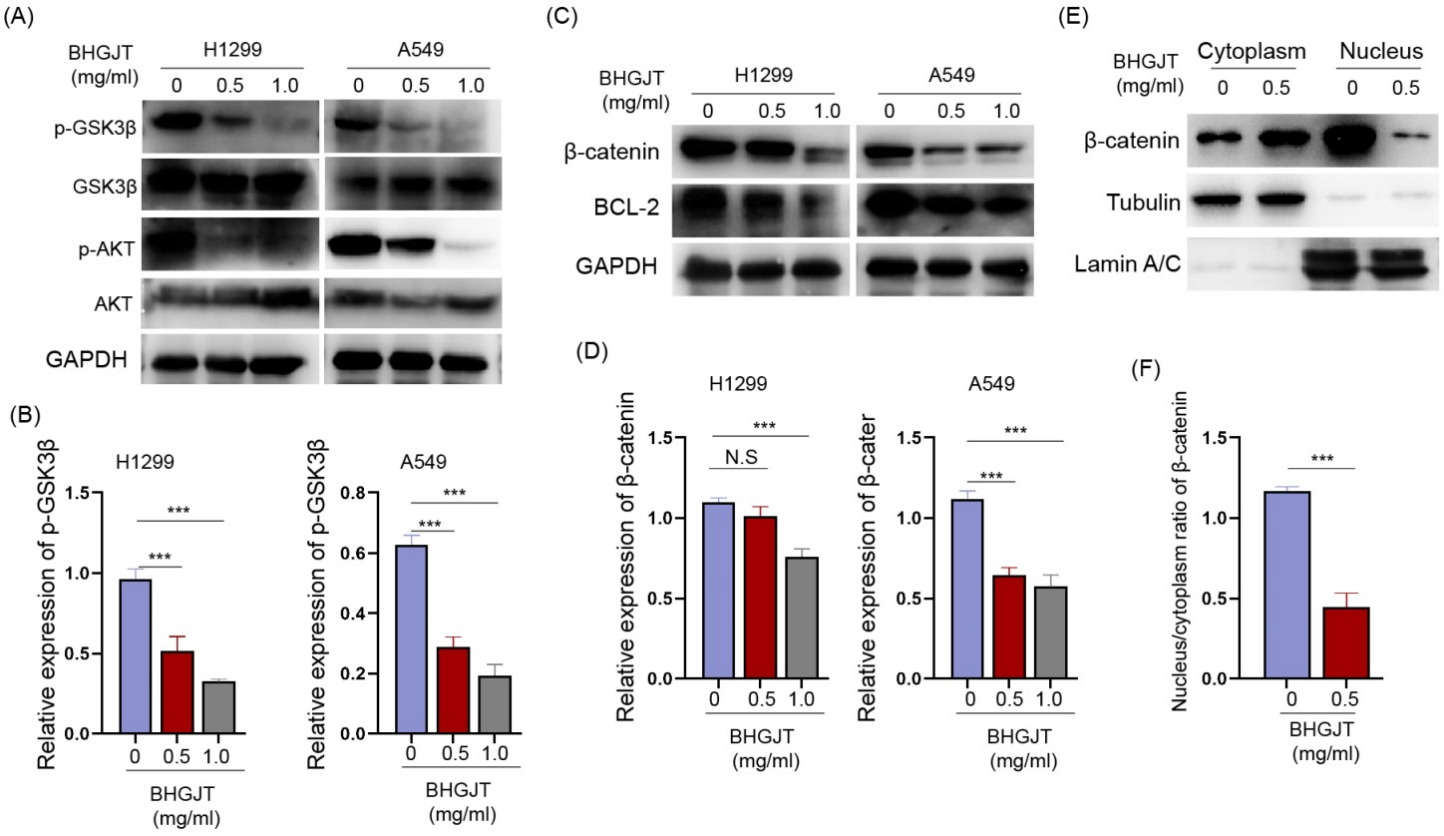
** BHGJT induces apoptosis by the AKT/GSK3β/β-catenin signaling pathway in lung cancer cells. (A,B)** Expression of p-GSK3β, GSK3β, AKT and p-AKT was detected by Western blot. BHGJT decreased the phosphorylation level of p-GSK3β and p-AKT upon stimulation with BHGJT in H1299 and A549 cells. **(C,D)** β-catenin was decreased by BHGJT, and downstream BCL-2 was also decreased in both cell lines. **(E,F)** Cytoplasmic and nuclear proteins were separately extracted. BHGJT significantly decreased the distribution of β-catenin in the nucleus compared to the control. *p<0.05; **p<0.01; ***p<0.001.

We detected the expression of AKT, p-AKT and its crucial downstream GSK3β in lung cancer cells upon treatment with BHGJT. GSK3β is constitutively activated, and AKT leads to its inactivation by phosphorylation of serine 9. p-AKT was significantly downregulated and p-GSK3β was downregulated by BHGJT in a concentration-dependent manner (Figure 6A,B). The active form of GSK3β promotes degradation of β-catenin and decreases the expression of the antiapoptotic protein BCL-2. As expected, β-catenin and BCL-2 were significantly inhibited by BHGJT in H1299 and A549 cells (Figure 6C,D). Furthermore, β-catenin in the nucleus was almost eliminated by BHGJT, which explained why BCL-2 was downregulated in lung cancer cells (Figure 6E,F). Thus, these results indicated that the AKT/GSK3β/β-catenin signaling pathway was at least partially responsible for BHGJT-mediated apoptosis in lung cancer cells.

### BHGJT induces autophagy by the AMPK/mTORC1/ULK1 signaling pathway in lung cancer cells

**Figure 7:**
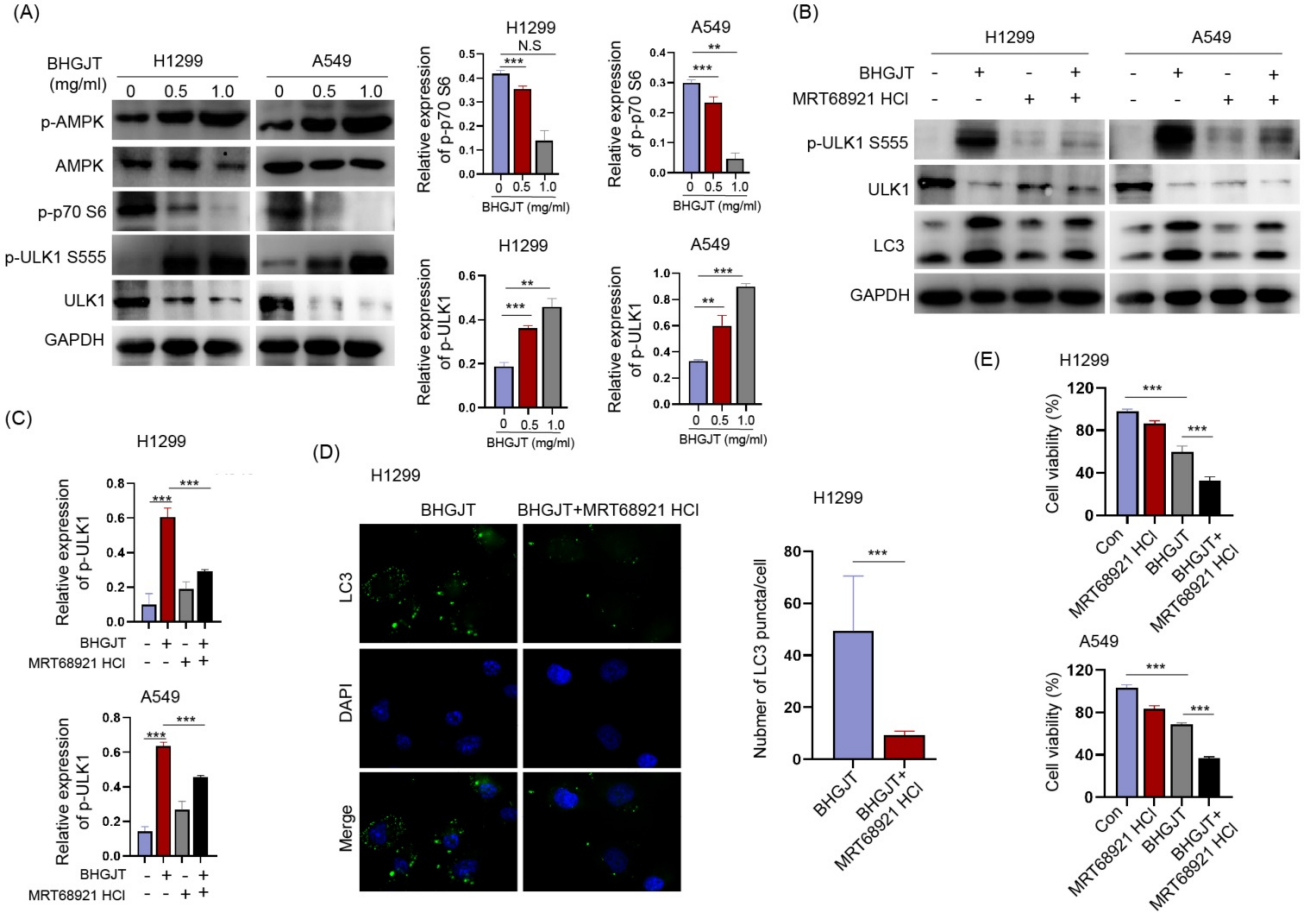
** BHGJT induces autophagy by the AMPK/mTORC1/ULK1 signaling pathway in lung cancer cells. (A)** Expression of p-AMPK, AMPK, p-p70 S6, p-ULK1 S555, and ULK1 was detected by Western blot. p-AMPK, and p-ULK1 S555 were significantly upregulated by BHGJT in a dose-dependent manner in H1299 and A549 cells, while p-p70 S6 was decreased by BHGJT. **(B,C)** MRT68921 is specific inhibitor of ULK1. Coadministration of MRT68921 and BHGJT led to similar expression of p-ULK1 S555 compared to the BHGJT group. Inhibition of ULK1 markedly decreased the expression of LC3-II upon stimulation with BHGJT in both cell lines. **(D)** As shown by immunofluorescent staining of LC3, MRT68921 significantly reduced the number of LC3 puncta induced by BHGJT in H1299 cells. **(E)** Inhibition of autophagy by a ULK1 inhibitor significantly sensitized lung cancer cells to BHGJT treatment. Con, control; **p<0.01; ***p<0.001.

Autophagy was induced by BHGJT in lung cancer cells; thus, we explored the underlying molecular mechanism. The AMPK/mTORC1/ULK1 signaling pathway is an essential regulator of autophagy activity. Western blot analysis showed that p-AMPK and p-ULK1 were significantly upregulated, p-p70 was downregulated by BHGJT in H1299 and A549 cells (Figure 7A). MRT68921 is a specific inhibitor of ULK1. Treatment with MRT68921 (5 nM) led to significant inhibition of p-ULK1 and decreased expression of LC3-II in both cell lines upon stimulation with BHGJT (Figure 7B,C). This result was further verified by immunofluorescent staining, which showed that treatment with MRT68921 significantly reduced the number of LC3 puncta in H1299 cells (Figure 7D). Inhibition of autophagy by MRT68921 markedly sensitized lung cancer cells to BHGJT compared to the control (Figure 7E) and resembled the result of CQ. Taken together, these results showed that BHGJT induced autophagy via the AMPK/mTORC1/ULK1 signaling pathway in lung cancer.

## Discussion

Herbal formulas such as BHGJT have been shown to exert a robust anticancer effect over a long time; however, the exact molecular mechanism underlying their effect is lacking. In the present work, we demonstrated that BHGJT inhibited lung cancer growth *in vitro* and *in vivo* by inducing cell cycle arrest and apoptosis and the anticancer effect could be further elevated by blocking autophagy. Regarding the mechanism, the AKT/GSK3β/β-catenin signaling pathway was responsible for growth arrest and apoptosis induced by BHGJT, and activation of autophagy was attributed to the AMPK/mTORC1/ULK1 signaling pathway in lung cancer cells. Therefore, our results suggested that BHGJT might be a potential effective complementary and adjuvant therapy for lung cancer.

Cancer has been recorded in ancient medical literature, and TCM represents the only treatment for those patients in China 19. From a modern medicinal perspective, TCM is commonly used to improve the efficiency of chemotherapy, radiotherapy, or immunotherapy and reduce adverse effects in Chinese people around the world. Unlike molecular-targeted Western medicine, which usually applies single or several chemical components, TCM relies on an integrative approach involving multiple herbs in one formula 20. Several TCM formulas have been demonstrated to efficiently kill lung cancer cells *in vitro* and inhibit tumor growth *in vivo*. For instance, Lian-Jia-San-Jie-Fang (LJSFJ) significantly reduces the number of colonies formed and the growth of tumors by regulating the EGFR and p53 signaling pathways in lung cancer cells 21. Ze-Qi-Tang (ZQT), another TCM consisting of 9 herbs, induces apoptosis by a p53-dependent pathway and cell cycle arrest by downregulating Cyclin B1 and Cdk2 in lung cancer cells and animal models 17. Tien-Hsien Liquid shows immunomodulatory activity and induces apoptosis in a wide variety of cancer cells 22. Jin formula reduces tumor growth through microRNA-dependent Wnt/β-catenin signaling in lung cancer cells and animal models 23. Our study provided evidence that BHGJT could directly inhibit lung cancer cells by dysregulating cell cycle-associated genes, such as CDK4 and Cyclin D1, and apoptosis-associated genes, such as Bax, Bad, BCL-2 and caspase 3. Our network pharmacology-based analysis also suggested that BHGJT-induced apoptosis relied on p53 signaling. However, the concentrations of TCM extract that show effectiveness against cancer cells are usually up to 0.5 or 1.0 mg/ml, although an obvious difference might be observed between the water extract and ethanol extract of the same formula. Thus, TCM is not commonly used as a single treatment for cancer patients.

A basic hypothesis is that a properly formulated herbal cocktail might target multiple cellular pathways to obtain synergistic or interactive effects on various targets or dysfunctional bioprocesses in cancers 19, 24-26. Thus, the use of TCM can lead to a considerably enhanced anticancer effect compared to the use of a single herb or may provide a synergistic therapeutic effect along with conventional therapy in patients with cancer. For example, the cytotoxic effect of LJSJF on lung cancer cells is elevated considerably compared to that of the single herbs involved in LJSJF 21. Tien-Hsien Liquid possesses antimetastasis capacity by decreasing MMP-2, MMP-9 and uPA expression and tumor growth and promoting antiangiogenic effects 22. For combination therapy, chemotherapy combined with TCM herbal treatment has beneficial effects on improving quality of life and patient 1-, 2-, or 3-year survival in lung cancer 27-29. The combination of targeted therapy with TCM shows a higher objective response rate than targeted therapy alone in non-small cell lung cancer 30. A large-scale study showed that TCM provides a significant protective effect for patients with lung cancer and leads to a 32% reduction in all-cause mortality 27. Based on our findings, inhibition of autophagy largely augmented the toxic effect of BHGJT on lung cancer cells. The role of several autophagy inhibitors for the treatment of cancers, such as hydrochloroquine or CQ, has been severely underestimated 31. Thus, exploring the combination therapy of BHGJT with other therapies would provide greater benefits for patients with lung cancer.

In summary, we demonstrated that BHGJT efficiently inhibits tumor growth *in vitro* and in animal models and verified that BHGJT induced pronounced cell cycle arrest at the G0/G1 phase by downregulating CDK4 and Cyclin D1 and promoted apoptosis by a mitochondria-dependent pathway via AKT/GSK3β/β-catenin signaling. Additionally, we revealed that protective autophagy was obviously induced by BHGJT via the AMPK/mTORC1/ULK1 signaling pathway and that inhibiting autophagy by CQ or MRT68921 facilitated cell death upon treatment with BHGJT in lung cancer. This work provides preclinical evidence demonstrating the anticancer efficacy of BHGJT and suggests that it is appropriate for use as a complementary therapy or for incorporation in combination therapy for lung cancer patients.
